# Relationships between trait and respiratory parameters during quiet breathing in normal subjects

**DOI:** 10.1007/s12576-017-0539-7

**Published:** 2017-05-02

**Authors:** Akae Kato, Koki Takahashi, Ikuo Homma

**Affiliations:** Department of Judo Therapy, Tokyo Ariake University of Medical and Health Sciences, 2-9-1 Ariake Koto-ku, Tokyo, 135-0063 Japan

**Keywords:** STAI, Respiratory rate, Trait anxiety, Quiet breathing

## Abstract

Respiratory patterns are influenced and altered by various emotional changes. In the present study, we investigated how respiratory patterns differ from individual to individual during quiet breathing. We examined the State-Trait Anxiety Inventory and various respiratory parameters in 16 healthy male subjects. Tidal volume was significantly larger and respiratory rate (RR) was significantly higher in both the higher trait (HT) and higher state (HS) anxiety groups compared to the lower trait and lower state anxiety groups. Inspiratory (*T*_I_) and expiratory time (*T*_E_) was significantly shorter in both the HT and HS anxiety groups. There was no significant difference in minute ventilation between these two groups. End-tidal CO_2_%, heart rate, and oxygen uptake ($\dot{\rm {V}} {\rm{O_2}}/W$) also showed no significant differences. *V*_T_ showed a negative correlation and RR showed a positive correlation with trait scores. *T*_I_ and *T*_E_ showed a negative correlation with trait anxiety scores. However, no other respiratory parameter showed any correlation. These results suggest that the respiratory rhythm reflected by RR is affected by the activity generated in the higher center in accordance with the level of trait anxiety during quiet breathing in awake humans.

## Introduction

It is well known that the main function of breathing is to inhale oxygen and expire carbon dioxide for maintaining life. The breathing of this function is called metabolic breathing and is generated in the respiratory center located in the brainstem, particularly in the medulla and pons. However, in addition to metabolic breathing, the so-called behavioral breathing is generated in the upper center in the brain. Behavioral breathing is generated by various internal or external environmental changes. A subtype of behavioral breathing, which is influenced by various emotions, has recently been investigated and has been termed, “emotional breathing” [1]. Therefore, autonomic breathing output generated by the combination of tidal activity and respiratory rhythm is not only controlled by metabolic demands but also controlled by constantly responding changes of emotions, such as fear, anxiety, sadness, and happiness. It is interesting that maintenance of both homeostasis and emotions coexist in the breathing pattern.

The center for emotional breathing may be in the limbic system, especially in the amygdala, which is well known as the primary center for emotions [2, 3]. It has been shown in an animal experiment that spontaneous burst activities recorded in the amygdala are functionally coupled to medullary respiratory rhythm in the limbic-brainstem-spinal cord preparation of a newborn rat. Electrical stimulation applied to the amygdala induces an inspiratory burst in the root of the phrenic nerve [4]. Spontaneous respiratory rhythmic activities are increased by the local application of CRF (corticotrophin releasing factor) in the preparation [5]. CRF is well known to integrate the global mammalian stress response [6, 7]. The relationship of respiratory activity and emotion has been shown in a study on anticipatory anxiety [8]; the subject’s respiratory rate increased during the feeling of anticipatory anxiety. In an earlier study, the changes in respiratory rate were found to be positively correlated with individual trait anxiety scores, and the amygdala was examined to determine the source generating the activity using a neuroimaging method during a time of anticipatory anxiety experienced by the subject [9].

Concomitant changes in breathing patterns and emotions have been shown in studies of odor stimulation and in the performance of Ikebana, the Japanese traditional art of flower arrangement. Unpleasant odor stimulation increased the respiratory rate and state anxiety scores, whereas they were decreased by pleasant odor stimulation [10]. Subjects showed a decrease in the state anxiety score and respiratory rate after performing Ikebana. The effects were more significant in subjects with a high trait anxiety [11]. Several previous studies have examined breathing patterns not only in normal subjects but also in subjects with anxiety disorders and found that the breathing patterns were linked to psychosomatic complains [12, 13].

A previous study of Masaoka and Homma (1997) showed that mental stimulation decreases expiratory time and increases minute ventilation. They also found a negative correlation between expiratory time and anxiety scores during the mental stimulation [14].

It is interesting to consider whether these two parameters, breathing pattern and anxiety scores, have a close relationship and also whether that relationship is different in different individuals during quiet breathing. The present study measured and compared breathing patterns and trait anxiety scores during quiet breathing in normal young adult males.

## Methods

### Subjects

Sixteen healthy male subjects aged from 19 to 23 years (20.8 ± 1.1 mean ± SD) participated in this study. No subjects had a psychiatric, neurological or pulmonary disorder. All subjects provided written informed consent, and the study was approved by the Ethics Committee of Tokyo Ariake University of Medical and Health Sciences.

### Procedure and measurements

Subjects sat on a chair wearing a facemask with a transducer connected to a respiratory monitor (AE-100i, Minato Medical Co., Ltd., Osaka) for measuring respiratory pattern and metabolism in a quiet room. Heart rate was also measured by a pulse oximeter (BSM-2401, NIHON KOHDEN Co., Ltd., Tokyo). After the subjects remained quiet for 3 min, respiratory rate per minute (RR), minute ventilation ($\dot{V}_{\rm E}$), tidal volume (*V*_T_), inspiratory time (*T*_I_), expiratory time (*T*_E_), end-tidal CO_2_% (FetCO_2_) and oxygen consumption per weight ($\dot{\rm {V}} {\rm{O_2}}/W$) was measured breath by breath for 5 min. All respiratory data with heart rate (HR) were stored on a laptop computer. The room temperature was maintained at 22.5 ± 1 °C.

### Psychological measurements

The anxiety level of each subject was accessed using Spielberger’s State-Trait Anxiety Inventory (STAI) [15]. The instrument comprises two scales, one for measuring Trait, and one for measuring State anxiety level. Each scale has 20 statements and anxiety levels for subjects are indicated by scores rating from 20 to 80. The trait anxiety score evaluates how people feel generally, while the state anxiety score evaluates how people feel ‘right now’ in various situations. Trait score is generally stable, while state score changes depending on the situation [15]. Trait scores of more than 44 indicate a high trait anxiety, and scores of less than 43 reflect normal or low trait anxiety in men. State scores of more than 41 indicate high state anxiety, and scores of less than 40 reflect normal or low trait anxiety in men. In this study, subjects were asked to assess their anxiety level using STAI before the start of physiological measurements.

Subjects were divided into two groups based on trait anxiety according to Spielberger’s State-Trait Anxiety Inventory (STAI): higher trait anxiety (HT) group and lower trait anxiety (LT) group. Trait anxiety score of the HT subjects was greater than 44 and the score in the LT subjects was less than 43. Subjects were also divided into two groups according to state anxiety: higher state anxiety (HS) group (more than 41) and lower state anxiety (LS) group (less than 40).

### Data analysis

All statistical analyses were performed with commercially available statistical package (JMP Pro13.0.0; SAS Institute Inc., Cary, NC, USA). Comparisons of all respiratory parameters with HR between HT and LT groups and between HS and LS were analyzed using unpaired *t* test and non-parametric unpaired Mann–Whitney test. As the results were similar, the latter results are presented.

Continuous valuables of the trait and state anxiety scores, which were not normally distributed, are shown as median and interquartile ranges. A *p* value of <0.05 was considered statistically significant. Spearman’s nonparametric correlation coefficients (*ρ*) were used to evaluate whether the trait or state anxiety score was correlated with any of the various respiratory parameters.

## Results

### STAI (Trait and State Anxiety)

**Fig. 1:**
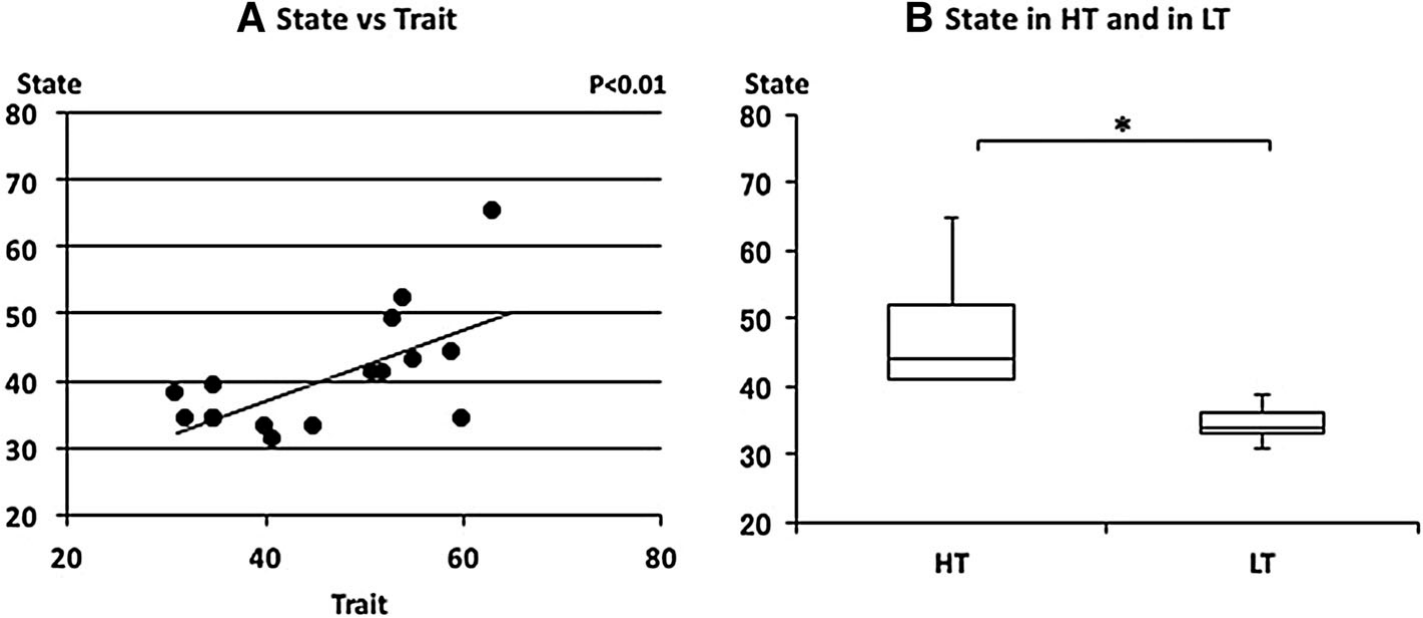
The relationship between state and trait anxieties. **a** Linear plot of state and trait scores in normal subjects. A significant positive correlation was observed (*ρ* = 0.580, *p* < 0.01). **b** Comparison of state scores in the high trait anxiety group (HT) and in the low trait anxiety group (LT). State scores were significantly higher in HT than in LT (*p < 0.05). Median state anxiety in HT and LT were indicated with *horizontal bars*. *The vertical bars* indicate the range and the *horizontal boundaries of the boxes* represent the first and third quartiles

Trait and state anxiety scores with the age of each subject is shown in Table 1. Trait anxiety scores varied from 31 to 63 and the median score and interquartile range was 48.0 (35.0–54.8) (median, 25th–75th percentiles). State anxiety scores also varied from 31 to 65 and the median score was 38.5 (34.0–43.8). Scores of the trait and state anxiety score were relatively similar for each subject and a positive correlation was observed between trait and state anxiety scores (*p* < 0.01) (Fig. 1a). The median of the state scores was 44.0 (41.0–52.0) and 34.0 (33.0–36.0) in HT and LT, respectively (Fig. 1b). The state scores were significantly higher in HT subjects (*p* = 0.019).

**Table 1:** STAI scores with the age of each subject

Subject (No.)	Age (year)	STAI	
		Trait	State
1	20	63	65
2	21	54	52
3	23	35	34
4	22	59	44
5	20	55	43
6	21	51	41
7	21	32	34
8	21	45	33
9	21	52	41
10	20	60	34
11	20	41	31
12	23	35	34
13	21	35	39
14	20	53	49
15	20	40	33
16	19	31	38
	20.8 ± 1.1	48.0 (35.0–54.8)	38.5 (34.0–43.8)
	Mean ± SD	Median (25th–75th percentiles)

Relationships between respiratory parameters, HR and $\dot{\rm {V}} {\rm{O_2}}/W$ with trait and state anxiety

**Fig. 2:**
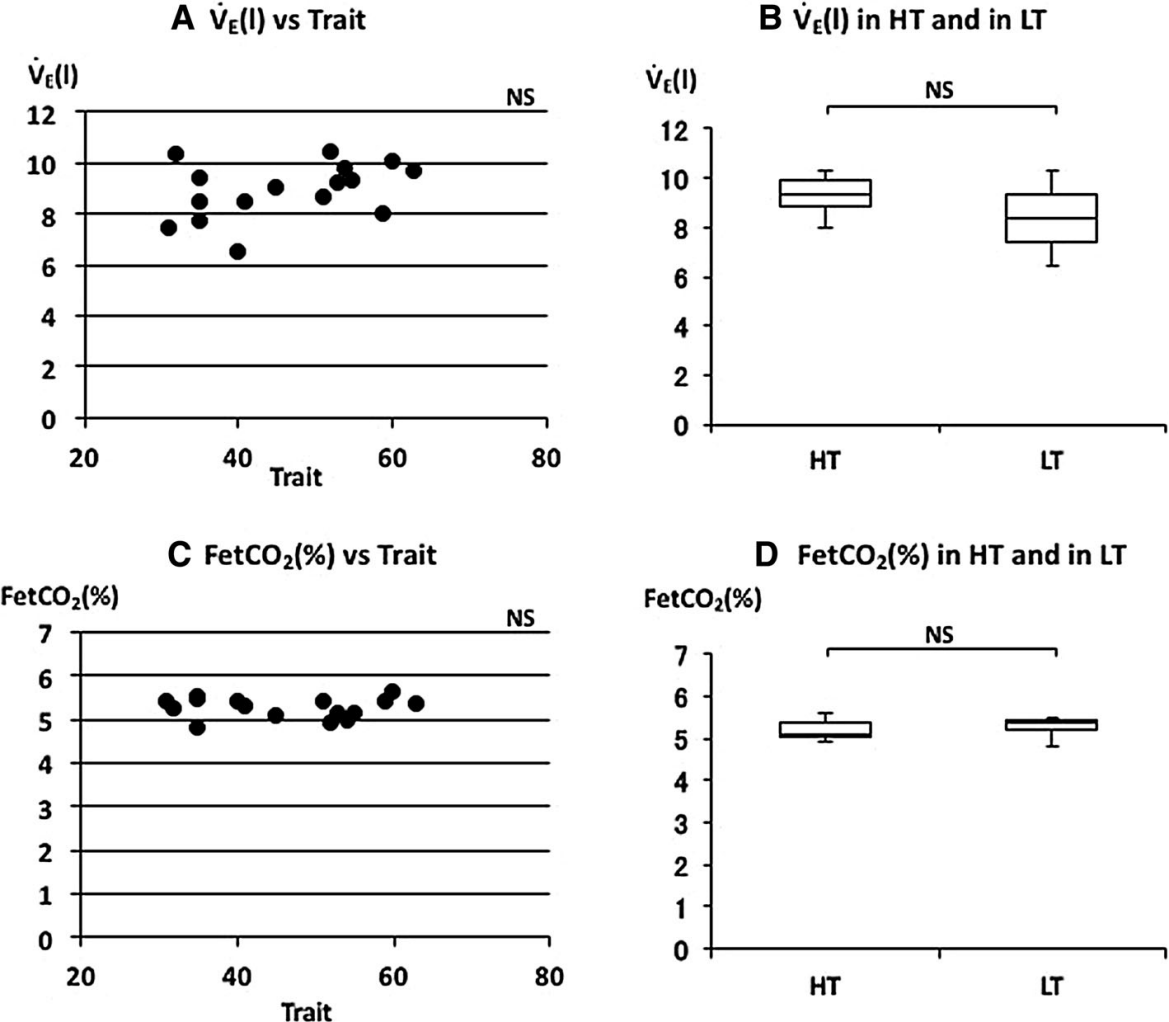
The relationships between minute ventilation ($\dot{V}_{\rm E}$), end-tidal CO_2_% (FetCO_2_), and trait anxiety. **a** Linear plot of $\dot{V}_{\rm E}$ and trait scores. No significant correlation was observed (*ρ* = 0.384). **b** Comparison of $\dot{V}_{\rm E}$ in HT and in LT. No significant difference was observed in $\dot{V}_{\rm E}$. **c** Linear plot of FetCO_2_ and trait scores. No significant correlation was observed (*ρ* = −0.038). **d** Comparison of FetCO_2_ in HT and in LT. No significant difference was observed in FetCO_2_. Median $\dot{V}_{\rm E}$ and FetCO_2_ in HT and LT were indicated with *horizontal bars*. *The vertical bars* indicate the range and *the horizontal boundaries of the boxes* represent the first and third quartiles

**Fig. 3:**
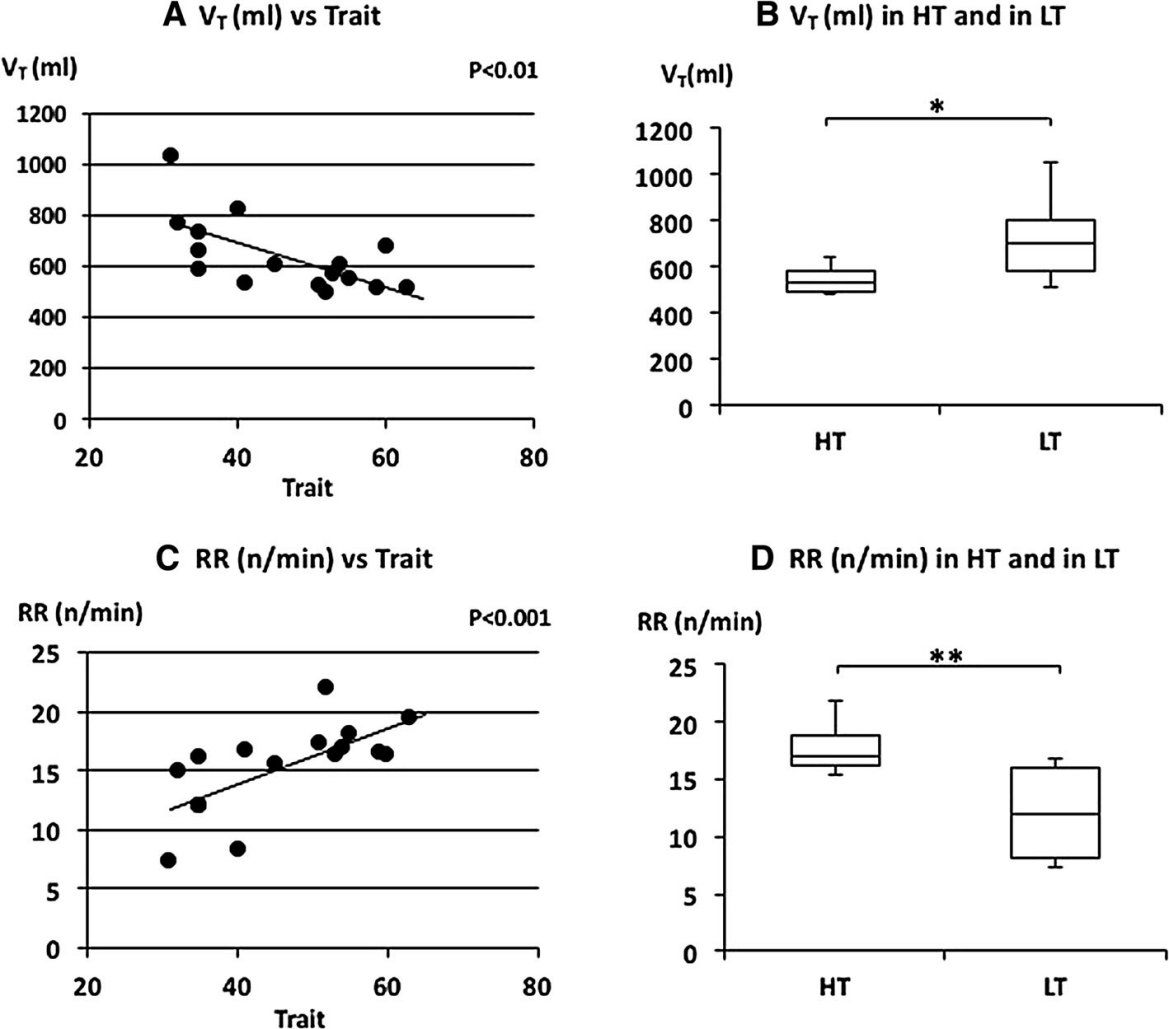
The relationships between tidal volume (*V*_T_), respiratory rate (RR) and trait anxiety. **a** Linear plot of *V*_T_ and trait scores. A significant negative correlation was observed (*ρ* = −0.687, *p* < 0.01). **b** Comparison of *V*_T_ in HT and in LT. *V*_T_ in LT was significantly larger than in HT (*p < 0.05). **c** Linear plot of RR and trait scores. A significant positive correlation was observed in RR (*ρ* = 0.749, *p* < 0.05). **d** Comparison of RR in HT and in LT. RR in HT was significantly higher than in LT (**p < 0.01). Median *V*_T_ and RR in HT and LT were indicated with *horizontal bars*. *The vertical bars* indicate the range and *the horizontal boundaries of the boxes* represent the first and third quartiles

**Fig. 4:**
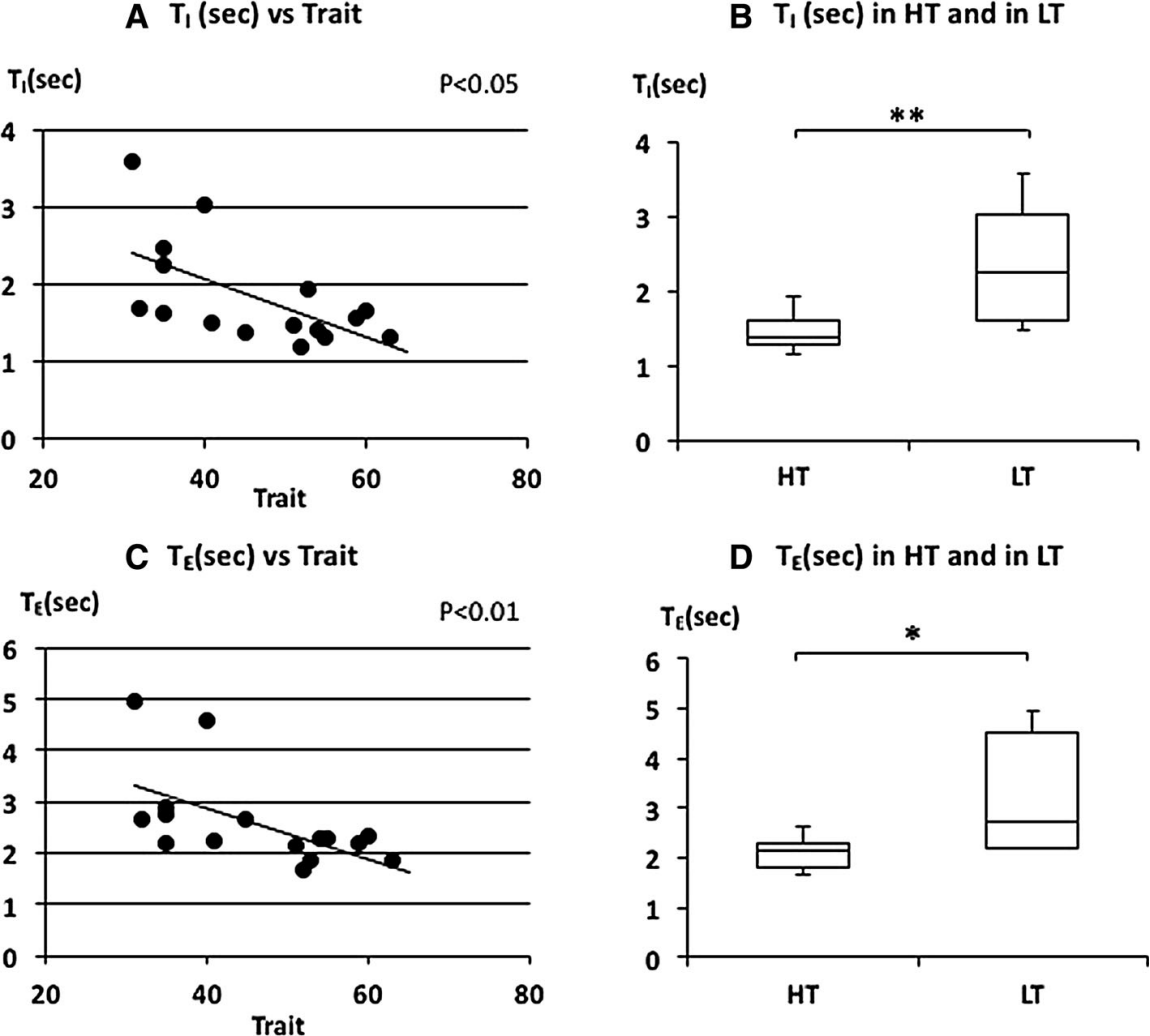
The relationship between inspiratory time (*T*_*I*_), expiratory time (*T*_E_), and trait anxiety. **a** Linear plot of *T*_I_ and trait scores. A significant negative correlation was observed in *T*_I_ (*ρ* = −0.622, *p* < 0.05). **b** Comparison of *T*_I_ in HT and in LT. *T*_I_ in HT was significantly shorter than in LT (**p < 0.01). **c** Linear plot of *T*_E_ and trait scores. A significant negative correlation was observed (*ρ* = −0.631, *p* < 0.05). **d** Comparison of *T*_E_ in HT and in LT. *T*_E_ in HT was significantly shorter than in LT (*p < 0.05). Median *T*_I_ and *T*_E_ in HT and LT were indicated with *horizontal bars*. *The vertical bars* indicate the range and *the horizontal boundaries of the boxes* represent the first and third quartiles

**Fig. 5:**
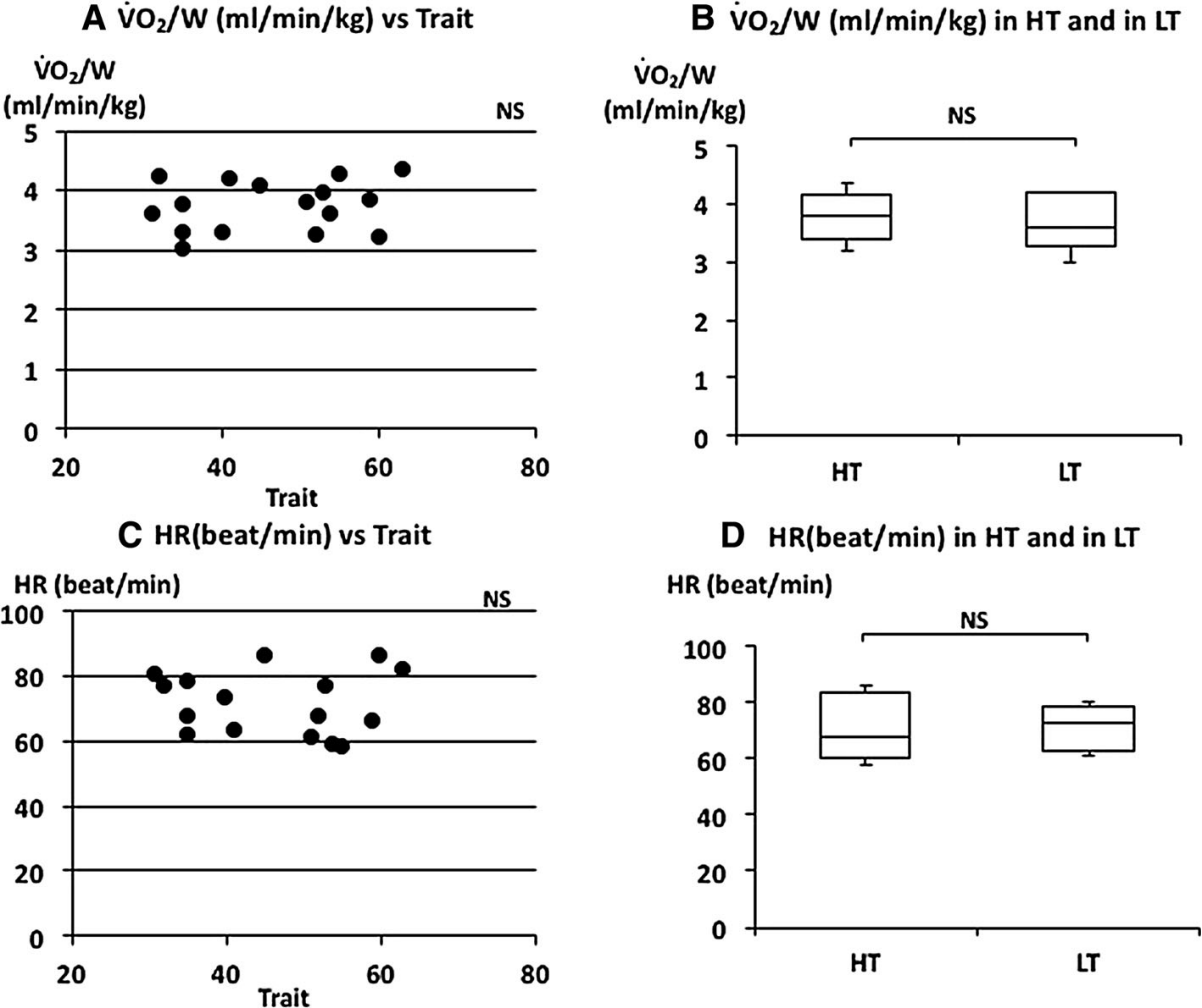
The relationship between oxygen uptake ($\dot{\rm {V}} {\rm{O_2}}/W$), heart rate (HR), and trait anxiety. **a** Linear plot of $\dot{\rm {V}} {\rm{O_2}}/W$ and trait scores. No significant correlation was observed (*ρ* = 0.209). **b** Comparison of $\dot{\rm {V}} {\rm{O_2}}/W$ in HT and in LT. No significant difference was observed in $\dot{\rm {V}} {\rm{O_2}}/W$. **c** Linear plots of HR and trait scores. No significant correlation was observed (*ρ* = −0.047). **d** Comparison of HR in HT and in LT. No significant difference was observed in HR. Median $\dot{\rm {V}} {\rm{O_2}}/W$ and HR in HT and LT were indicated with *horizontal bars*. *The vertical bars* indicate the range and *the horizontal boundaries of the boxes* represent the first and third quartiles

The relationships between the various respiratory parameters, FetCO_2_, $\dot{V}_{\rm E}$, *V*_T_, RR, *T*_I_, *T*_E_, or $\dot{\rm {V}} {\rm{O_2}}/W$ and STAI scores were examined. There was no significant correlation between FetCO_2_(%) and trait anxiety scores (Fig. 2c). There was no significant correlation between $\dot{V}_{\rm E}$ and trait anxiety scores (Fig. 2a). $\dot{V}_{\rm E}$ is determined by the combination of RR and *V*_T_. *V*_T_ was negatively and RR was positively correlated with trait anxiety scores (Fig. 3a, c). RR is based on inspiratory time (*T*_I_) and expiratory time (*T*_E_). *T*_I_ and *T*_E_ were also negatively correlated with trait anxiety scores (Fig. 4a, c). There was no significant correlation between trait anxiety scores and HR or $\dot{\rm {V}} {\rm{O_2}}/W$ (Fig. 5a, c). *V*_T_ and *T*_E_ were negatively and RR was positively correlated with state anxiety scores (Table 2). Correlations between HR or $\dot{\rm {V}} {\rm{O_2}}/W$ and state anxiety scores were also examined. Neither parameter showed a significant correlation with state anxiety scores (Table 2).

**Table 2:** Correlation of respiratory parameters with trail (A) and (B)

A. Trait					
Circulation			Differences		
	*ρ*	*P*	HT	LT	*P*
$\dot{V}_{\rm E}$(I)	0.384	NS	9.3 (8.9–9.9)	8.4 (7.4–9.3)	NS
FetCO_2_ (%)	−0.038	NS	5.1 (5.0–5.4)	5.4 (5.2–5.4)	NS
*V*_t_ (ml)	−0.687	<0.01	537.0 (493.8–584.1)	699.2 (585.7–803.21)	<0.05
RR (n/min)	0.749	<0.001	16.9 (6.3–18..8)	12.1 (8.2–16.1)	<0.01
*T*_I_ (s)	−0.622	<0.05	1.4 (1.3–1.6)	2.2 (1.6–3.0)	<0.01
*T*_E_ (s)	−0.631	<0.01	2.2 (1.8–2.3)	2.7 (2.2–4.5)	<0.05
$\dot{\rm {V}} {\rm{O_2}}/W$ (ml/min/kg)	0.209	NS	3.8 (3.4–4.2)	3.6 (3.3–4.2)	NS
HR (beat/min)	−0.047	NS	67.7 (60.0–83.8)	73.1 (62.8–78.5)	NS

B. State					
Circulation			Differences		
	*ρ*	*P*	HS	LS	*P*
$\dot{V}_{\rm E}$(I)	0.349	NS	9.3 (8.6–9.7)	8.47 (7.6–9.7)	NS
FetCO_2_ (%)	−0.319	NS	5.1 (5.0–5.4)	5.4 (7.6–9.7)	NS
*V*_t_ (ml)	−0.558	<0.05	498.9 (490.1–572.1)	655.2 (587.3–777.1)	<0.01
RR (*n*/min)	0.582	<0.05	17.2 (16.5–19.5)	15.0 (10.1–16.2)	<0.01
*T*_I_(s)	−0.404	NS	1.4 (1.3–1.5)	1.7 (1.5–2.7)	<0.05
*T*_E_ (s)	−0.623	<0.01	2.1 (1.8–2.2)	2.6 (2.3–3.7)	<0.01
$\dot{\rm {V}} {\rm{O_2}}/W$ (ml/min/kg)	0.212	NS	3.8 (3.6–4.3)	3.6 (3.2–4.1)	NS
HR (beat/min)	−0.245	NS	65.8 (58.8–76.6)	76.4 (65.2–83.3)	NS

Differences of respiratory parameters between high (HT) and low (LT) trail and high (HS) and low (LS) state

### Comparisons of respiratory parameters, HR and $\dot{\rm {V}} {\rm{O_2}}/W$ in HT, LT and HS, LS groups

The median of FetCO_2_(%) was 5.11 (5.01–5.39) in HT and 5.36 (5.22–5.42) in LT (Fig. 2d). There was no significant difference in FetCO_2_(%) (*p* = 0.397). The mean of $\dot{V}_{\rm E}$ was 9.3 (8.81–9.88) L/min in HT and 8.39 (7.41–9.32) L/min in LT (Fig. 2b). There was no significant difference in $\dot{V}_{\rm E}$ (*p* = 0.081). The median of *V*_T_ was 536.95 (493.78–584.15) ml in HT and 699.15 (585.68–803.07) ml in LT subjects (Fig. 3b). The *V*_T_ was significantly larger in LT subjects (*p* = 0.011). The median RR was 16.92 (16.25–18.75) n/min in HT and 12.06 (8.23–16.09) n/min in LT subjects (Fig. 3d). The RR was significantly higher in HT subjects (*p* = 0.005). The median of *T*_I_ and *T*_E_ were 1.38 (1.29–1.59) and 2.15 (1.82–2.27) s in HT and 2.23 (1.59–3.02) and 2.71 (2.22-4.54) s in LT subjects, respectively (Fig. 4b, d). *T*_I_ and *T*_E_ were significantly shorter in HT subjects (*p* = 0.008 for *T*_I_ and *p* = 0.015 for *T*_E_). The median of $\dot{\rm {V}} {\rm{O_2}}/W$ was 3.82 (3.41–4.16) ml/W in HT and 3.61 (3.28–4.19) ml/W in LT subjects (Fig. 5b). There was no significant difference in $\dot{\rm {V}} {\rm{O_2}}/W$ (*p* = 0.459). The median of HR was 67.71 (60.00–83.75) beat/min in HT and 73.10 (62.77–78.54) beat/min in LT subjects (Fig. 5d). There was also no significant difference in HR between the two groups (*p* = 1.000). There were also no significant differences in $\dot{V}_{\rm E}$, FetCO_2_(%), $\dot{\rm {V}} {\rm{O_2}}/W$ and HR in higher state anxiety (HS) and lower state anxiety (LS) groups (Table 2).

## Discussion

Trait and state anxiety scores were distributed from low to high among subjects. Trait anxiety score is generally unchangeable, but state anxiety score is changeable. As state anxiety is measured according to how the subject feels ‘right now’ in various situations, it is affected by various environments and mental factors. On the other hand, trait anxiety represents the subjective feeling in general [15]. However, both anxiety scores were closely related and the median state anxiety score of the HT group was significantly higher than that of the LT group in this study. A positive correlation was also obtained between trait and state anxiety scores.

### Relationships between respiratory parameters and STAI

It is generally agreed that breathing patterns are generated in the brainstem under metabolic demands. Minute ventilation ($\dot{V}_{\rm E}$) reflects the volume demand from the metabolic change. HR and $\dot{\rm {V}} {\rm{O_2}}/W$ are also changed by metabolic demands. There were no differences in $\dot{V}_{\rm E}$, HR and $\dot{\rm {V}} {\rm{O_2}}/W$ between the higher and lower trait anxiety groups (HT and LT) and between the higher state and lower state anxiety groups (HS and LS) (Figs. 2b, 5b, d; Table 2). There were also no significant correlations between these parameters and trait or state anxiety scores. This indicates that parameters changing with metabolic demands are independent of trait or state anxiety. Contrary to the so-called “metabolic breathing”, we have so-called “behavioral breathing” in which our breathing is influenced by internal or external environmental changes. Autonomic breathing is not only controlled by metabolic breathing, but also responds to changes in emotions, such as anxiety, fear, and pleasantness. More rapid breathing, shown during an arousal state and during various negative emotional changes, indicates the relationships between emotions and respiration [14, 16–18]. Masaoka and Homma (2001) showed, in a study of anticipatory anxiety, that respiratory rate increases during anticipatory anxiety and that the response is not related to changes in metabolic demand [8]. They also showed that increased respiratory rate during anticipatory anxiety shows a linear positive correlation with trait anxiety scores. It is well known that subjects with idiopathic hyperventilation or panic disorder have high trait anxiety and increase their ventilation, which induces sustained arterial and alveolar hypocapnia [12, 19]. There was no hyperventilation observed in the present healthy subjects, even in those who had higher trait anxiety during quiet breathing. Irregular breathing, such as that described in subjects with anxiety or panic disorder [12, 20], was not apparent in these subjects. In the present study, we showed that the respiratory rate during quiet breathing is influenced by trait anxiety. Subjects who have a higher trait anxiety showed a higher respiratory rate and those with a lower trait anxiety showed a lower respiratory rate (Fig. 3d). We also found a positive correlation between individual respiratory rate and the values of trait anxiety score (Fig. 3c). State anxiety showed significant correlations with *V*_T_, RR, and *T*_E_. However, these correlation coefficients were smaller than those in trait anxiety. This indicated that these respiratory parameters are more strongly correlated with trait.

Anxiety and other emotions are primarily generated in the amygdala [2, 3]. Previous studies, using functional neuroimaging methods, have shown the neuroanatomical correlates of negative emotions of fear and anxiety and have revealed that the amygdala plays a crucial role in the processing of these emotions. Recently, respiratory rhythmic neural activities were recorded from the piriform cortex and amygdala using a limbic brainstem-spinal cord preparation in newborn rats [4]. The activity was synchronized with the burst activities recorded from the spinal root for the phrenic nerve and was functionally coupled to medullary respiratory rhythm. It is generally believed that basic respiratory rhythm is generated in the brainstem. The respiratory central pattern generator (RCPG) has been shown to be located in the brainstem [21]. A neuroimaging study using fMRI in awake humans showed that the limbic/paralimbic-bulbar circuitry plays a significant role in emotional modulation of spontaneous breathing [22]. The recent work study of Kim et al. (2013) showed that stimulation of the bed nucleus of the stria terminals (BNST), which is known to influence physiological manifestations of anxiety, increases the respiratory rate in mice [23]. In humans, dipoles obtained during the anticipatory anxiety were synchronized with respiration and were found to be located in the amygdala [9].

Human and animal experiments on limbic and paralimbic areas suggested that emotional impact is not only associated in neural activities in the brainstem, but also in the limbic and paralimbic regions particularly in amygdala in the limbic system [1].

The strong close relationship between respiratory rhythm and trait anxiety was also shown here in the relationship between inspiratory time (*T*_I_) and expiratory time (*T*_E_). *T*_E_ may contribute to the determination of RR and indeed *T*_I_ has been shown to have no correlation with trait anxiety during mental stimulation even though *T*_E_ was correlated with trait anxiety [14]. However, in this study significant correlations between *T*_E_ or *T*_I_ and trait anxiety were shown with a correlation between RR and trait anxiety during quiet breathing. The respiratory rate, independent of metabolic breathing demands, may be affected by trait anxiety during quiet breathing in humans.

These basic research data obtained from normal subjects would be useful for explication of the clinical condition of the hyperventilation syndrome and anxiety disorders.
